# Diversity Temporal–Spatial Dynamics of Potato Rhizosphere Ciliates and Contribution to Nitrogen- and Carbon-Derived Nutrition in North-East China

**DOI:** 10.3390/plants12122260

**Published:** 2023-06-09

**Authors:** Weibin Zheng, Xiaodan Zheng, Yuqing Wu, Shaoyang Lv, Chang Ge, Xiang Wang, Qiuhong Wang, Jingjing Cui, Nanqi Ren, Ying Chen

**Affiliations:** 1 Key Laboratory of Biodiversity of Aquatic Organisms, Harbin Normal University, Harbin 150025, China; zwbpro1993@hrbnu.edu.cn (W.Z.); 18645660035@163.com (X.Z.); wyq_06090@163.com (Y.W.); lsy971215@163.com (S.L.); gechang0310@163.com (C.G.); 18814515016@163.com (X.W.); 2 State Key Laboratory of Urban Water Resource and Environment, School of Civil and Environmental Engineering, Harbin Institute of Technology (Shenzhen), Shenzhen 518055, China; 3 Crop Academy, Heilongjiang University, Harbin 150080, China; wangqiuhong119@126.com (Q.W.); 15252829455@163.com (J.C.)

**Keywords:** potato, rhizosphere microorganism, ciliates, nitrogen nutrition, carbon nutrition

## Abstract

Ciliates are an important component of the rhizosphere microorganism community, but their nutritional contribution to plants has not been fully revealed. In this paper, we investigated the rhizosphere ciliate community of potatoes during six growth stages, illustrated the spatial–temporal dynamics of composition and diversity, and analyzed the correlation between soil physicochemical properties. The contributions of ciliates to the carbon- and nitrogen-derived nutrition of potatoes were calculated. Fifteen species of ciliates were identified, with higher diversity in the top soil, which increased as the potatoes grew, while they were more abundant in the deep soil, and the number decreased as the potatoes grew. The highest number of species of ciliates appeared in July (seedling stage). Among the five core species of ciliates, *Colpoda* sp. was the dominant species in all six growth stages. Multiple physicochemical properties affected the rhizosphere ciliate community, with ammonium nitrogen (NH_4_^+^-N) and the soil water content (SWC) greatly influencing ciliate abundance. The key correlation factors of ciliates diversity were NH_4_^+^-N, available phosphorus (AP), and soil organic matter (SOM). The annual average contribution rates of carbon and nitrogen by rhizosphere ciliates to potatoes were 30.57% and 23.31%, respectively, with the highest C/N contribution rates reaching 94.36% and 72.29% in the seedling stage. This study established a method for estimating the contributions of carbon and nitrogen by ciliates to crops and found that ciliates could be potential organic fertilizer organisms. These results might be used to improve water and nitrogen management in potato cultivation and promote ecological agriculture.

## 1. Introduction

The plant rhizosphere drives biological, chemical, and physical processes in soil to form an underground soil food web [1]. Plant and microorganisms constitute a relatively independent rhizosphere (a micro-ecological system) that impacts the decomposition and transformation of effective nutrients, the accumulation and stability of soil organic carbon, root morphology and physiology, nutrient absorption capacity, and multi-nutrient relationships in the aboveground parts of plants [2]. Meanwhile, plant growth and development affect the activity and community structure of the rhizosphere microbiota [3,4]. 

The protozoa are one of components of the rhizosphere micro-community, which can regulate microbial activity [5,6] and accelerate the circulation and transformation of organic matter as they have a higher evolutionary status in the food chain than bacteria and trophic-level microbiota [7]. The dynamics of protozoa are also correlated with plant growth and development [6,8,9]. Ciliates are considered to be the most complex and highly differentiated protozoa, characterized by a short generation time, rapid reproduction, and multiple nutritional types. They are widely distributed in soil and interact with bacteria, fungi, and other protists to form complex micro-food webs [10], playing a crucial role in the energy flow and material circulation in the soil ecosystem [10]. Recently, the importance of rhizosphere ciliates in soil carbon and nitrogen sources was explored in sugar beet [11], but more work needs to be carried out on other plants. The species, spatial and temporal distribution, ecological function, and mechanism of soil ciliates are key issues in the micro-ecological system. Potatoes (*Solanum tuberosum* L.) are the world’s fourth-largest food crop after rice, maize, and wheat, and it is a staple in the human diet in many places around the world. Some researchers have explored the key microorganisms of the potato rhizosphere, focusing on harmful pathogenic groups and the nutritional availability of inoculation groups [12]. In addition, the microbe–fertilizer–crop relationship was revealed by comparing the composition and structure of the potato rhizosphere microbial community and the potato growth state under different fertilization treatment conditions [13,14]. The authors of the studies suggested that bacteria can facilitate the conversion of organic matter into forms that are usable by plant roots, and fungi can break down complex organic compounds to release nitrogen and other essential nutrients [15,16,17,18]. However, the release of carbon and nitrogen via ciliate activity in potato production systems has not been well understood, and their interactions with other biotic and abiotic factors remain undisclosed.

In this paper, we studied the temporal–spatial features of potato rhizosphere ciliate communities across four soil layers and six growth stages in north-east China. The contribution of ciliates to the carbon- and nitrogen-derived nutrition of potatoes in every growth stage was estimated using a series of equations. The potential ability of ciliates in organic fertilizer and water and nitrogen management in potato cultivation were discussed.

## 2. Results

### 2.1. Composition and Diversity of Ciliates Community 

**Figure 1:**
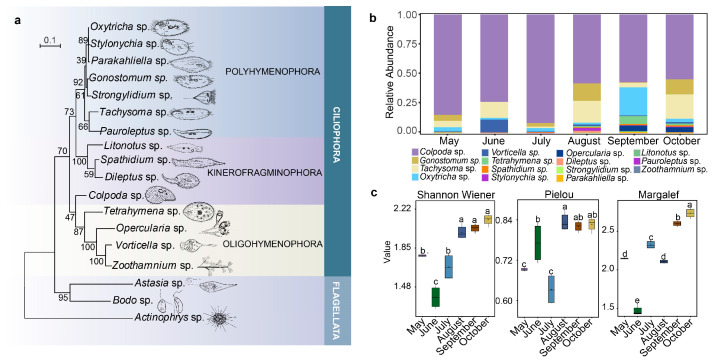
Ciliates community composition and diversity from May to October (**a**). Taxa and phylogeny of protozoa. (**b**) Relative abundance of culturable ciliates in six growth stages. (**c**) Diversity index of ciliates during six growth stages of potatoes. Box plots labelled with different letters represent statistically significant differences (*p* < 0.05).

The protozoa taxa of the potato rhizosphere were identified using the non-flooded Petri dish method and morphological identification (Figure A3). There were fifteen species of ciliates and three flagellate species (Figure 1a). The 15 ciliates species clustered into three branches with high confidence: Polyhymenophora (7 species), Kinerofragminophora (4 species), and Oligohymenophora (4 species). The abundance of ciliates showed a diminishing trend from May to October, but an exceptional increase appeared at the seedling stage (July) (Table A1). The relative abundance (Figure 1b) and cumulative contribution (Table A2) showed that *Colpoda* sp. was the most abundant species at every stage. 

The temporal dynamics of three diversity indices showed that ciliates’ diversity generally increased as the potatoes grew (except in May, the pre-planting stage), while the Pielou evenness index in July (seedling stage) and the Margalef species richness index in August (rooting stage) fell abnormally (Figure 1c).

### 2.2. Spatial–Temporal Dynamics of Ciliates

**Figure 2:**
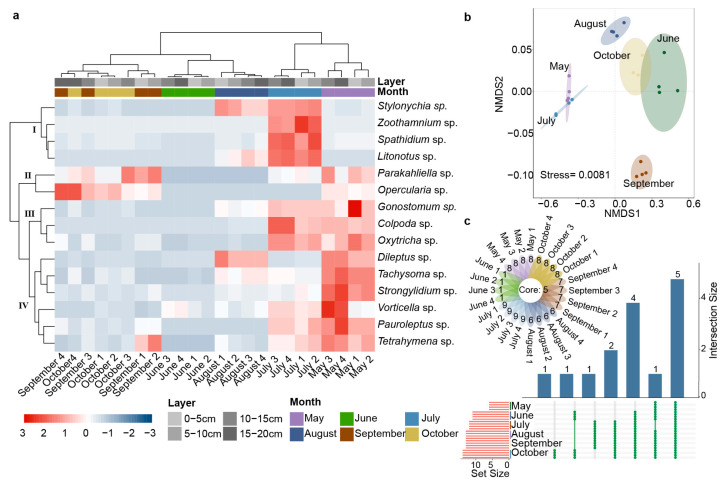
Spatial–temporal characteristics of potato rhizosphere ciliate communities: (**a**) the abundance and occurrence frequency of ciliates in four soil layers over six months; (**b**) NMDS analysis based on ciliate abundance; (**c**) spatial and temporal distribution of ciliate structure at different growth stages.

The abundance and occurrence frequency of the 15 ciliates over six months were differentiated into four clusters (I–IV) (Figure 2a). Six species in cluster IV showed the highest abundance value in May and July and the lowest abundance value in June. Four species in cluster I had higher abundance and occurrence frequencies in July. Cluster III contained three ciliates with the highest abundance value in May and July. Cluster II contained only two species with the highest abundance value in May, September, and October. According to the temporal characteristics of species and abundance, May and July were similar, while June and August were similar, and September was similar to October (Figure 2a).

During the growth stage of the potatoes, the diversity of ciliates in different soil layers was not significantly different. On the whole, the ciliate diversity of the top layer (0–10 cm) was always higher than that of the deep layer (10–20 cm) (Table A3), while the abundance of ciliates showed a continuous increase with the soil layer depth (Figure 2a).

Differences in ciliate community composition in the six growth stages were analyzed via NMDS (stress = 0.0081) (Figure 2b). The results showed that the ciliate communities in May and July were more similar, while those of June, August, and October were also similar. September was very different from other months. The number of ciliate species in different month was significantly different, with 14 species in July, but only 6 species in June. The spatial heterogeneity of ciliates in the four soil layers was not significant every month. There were five core ciliates that appeared every month and in every layer, including *Colpoda* sp., *Tachysoma* sp., *Oxytricha* sp., *Gonostomum* sp., and *Vorticella* sp. (Figure 2c). 

### 2.3. Correlation Analysis of Physicochemical Factors and Ciliate Community

Soil environmental factors have important effects on the rhizosphere microbial community. Eight soil physicochemical properties were analyzed to find out the key environmental factors for the potato rhizosphere ciliate community. The temperature decreased with the deepening of the layer soil and varied significantly during the seasons, with a range of 9–23 °C (Figure A2a). The variation in pH was insignificant, with a range of 6.08–7.55 (Figure A2c). The soil water content (SWC), except for the sudden increase due to rainfall in July, was relatively stable, ranging from 3.1 to 8.4%, and it gradually increased with increasing depth (Figure A2b). The concentrations of ammonium nitrogen (NH_4_^+^-N) and total nitrogen (TN) in September and October were higher than those in other months, but nitrate nitrogen (NO_3_^−^-N) showed the opposite trend (Figure A2f–h). The content of the soil organic matter (SOM) at the pre-planting stage was significantly higher than those at the other stages, owing to the application of organic fertilizer, while it decreased suddenly in July, which was attributable to the increase in precipitation (Figure A2e). The content of available phosphorus (AP) was slightly higher in May–July than it was in August–October, without significant differences between the different layers (Figure A2d). 

**Figure 3:**
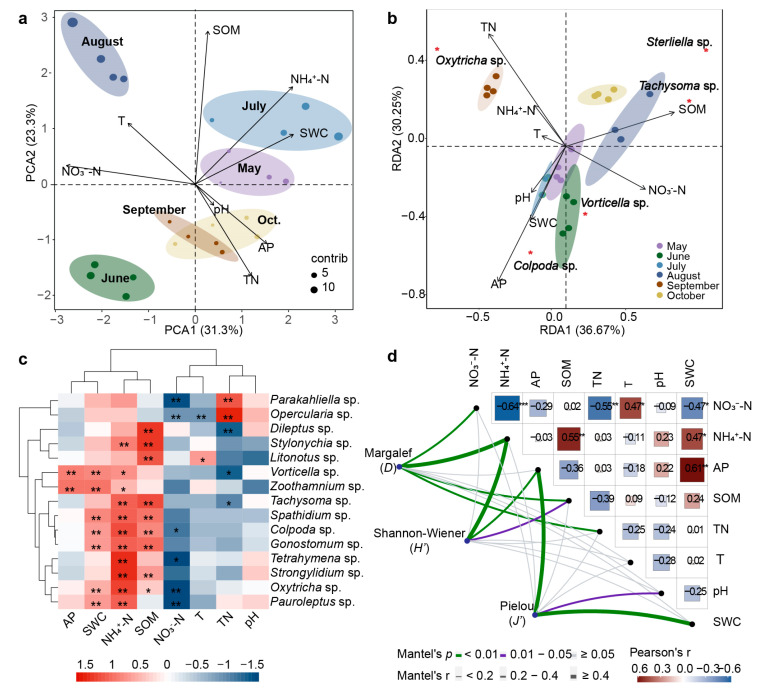
Correlation analysis between ciliates and physiochemical properties: (**a**) PCA plot based on log-transformed physicochemical data. Axes 1 and 2, respectively, account for 31.3% and 23.3% of the total variation; (**b**) RDA of principal coordinates on Bray–Curtis similarities from log-transformed species abundance data, and correlation between five highest contribution and community assembly dominant species with two RDA axes; (**c**) correlations (Spearman analysis) between abundance of 15 ciliates and soil properties; (**d**) Spearman analysis on soil properties and diversity indices of ciliates. *** Significant correlation at *p* < 0.001 level; ** Significant correlation at *p* < 0.01 level; * Significant correlation at *p* < 0.05 level.

The first two axes of the PCA plot account for 54.6% of the total variance in the dataset (Figure 3a). SOM, NH_4_^+^-N, and SWC were the top environmental factors at the pre-planting stage (May) and seedling stage (July). T and NO_3_^−^-N were the main environmental factors at the rooting stage (August), but pH, AP, and TN were key environmental factors at the mature stage (September) and late harvest stage (October). BIOENV analysis (Table A4) also indicated that multiple physicochemical properties can affect ciliate abundance via jointed interactions. According to the R value raking, the NH_4_^+^-N, SOM, TN, and pH combination had the highest correlation with the ciliate community. It was also notable that NH_4_^+^-N was the only variable included in all correlations.

The RDA plot (Figure 3b) shows the relationship between ciliate abundance and physicochemical factors in the six growth stages of potatoes. The model accounts for 66.92% of the total variation. The first ordination RDA axis explains 36.67% of the total variability in ciliate abundance, while the second ordination RDA axis explains 30.25%. The RDA plot also shows that the ciliate abundances at the same stage are clustered together, indicating a strong correlation between the potato growth stage and ciliate abundance. It should be noted that five core species were correlated with different environmental factors. *Colpoda* sp. and *Vorticella* sp. are correlated with pH, SWC, NO_3_^−^-N, and AP in May, June, and July, while *Terahymena* sp. and *Gonostomum* sp. are related to the variables SOM in September. *Oxytricha* sp. is related to T, NH_4_^+^-N, and TN in September. The Spearman correlation analysis also (Figure 3c) showed that NH_4_^+^-N and SWC greatly influenced the abundance of ciliates, followed by SOM, NO_3_^−^-N and TN, while AP had the smallest influence. *Colpoda* sp. and *Oxytricha* sp. are significantly positively correlated with NH_4_^+^-N, SWC, and SOM and are mainly negatively regulated by NO_3_^−^-N. *Tachysoma* sp. is positively correlated with SOM and NH_4_^+^-N, while it is negatively correlated with TN. *Gonostomum* sp. is significantly positively correlated with SWC, SOM, and NH_4_^+^-N. *Vorticella* sp. negatively regulates the soil TN, but positively regulates AP, SWC, and NH_4_^+^-N. Among the physicochemical properties affecting the abundance of non-dominant ciliate, soil SOM and NH_4_^+^-N are still the most influential properties, followed by NO_3_^−^-N, TN, and SWC. Mantel’s tests indicated that NH_4_^+^-N, AP, and SOM are the factors that are most strongly correlated with three diversity indices, and NO_3_^−^-N, TN, pH, and SWC are the factors that moderately affect the three diversity indices. Temperature is not a key factor affecting the three biodiversity indices (Figure 3d).

## 3. Discussion

### 3.1. Contribution of Rhizosphere Ciliates on Potato Growth

Carbon- and nitrogen-derived nutrition plays a crucial role in both vegetative and reproductive growth [19,20,21]. The contribution of rhizosphere microbial communities, especially bacteria and fungi, to carbon- and nitrogen-derived nutrition has been disclosed in many plants [17,22,23]. Researchers should be pay more attention to ciliate contributions to the carbon and nitrogen cycles, as they are essential components of the soil micro-food web and regulators of functional microorganisms that enhance C and N uptake and promote plant growth and soil structure formation [5]. Ciliate grazing can improve the carbon utilization efficiency of bacteria from 0.51 to 0.62 [24], and the rate of soil N mineralization (×1.8 in absence of plants) is doubled due to the activity of ciliates [25]. However, before now, an effective method to directly estimate the carbon and nitrogen contribution of ciliates was lacking. The authors of this study established a method for estimating the contribution of carbon and nitrogen by ciliates to crops based on the mechanism of grazing bacteria [25,26]. The results proved the great contribution of ciliates in competing for carbon and nitrogen sources during potato growth. Especially, the seedling stage (July) is a key stage, with the highest C/N contribution rates of 94.36% and 72.29% (Table 1). The corresponding estimated mean value for N released due to bacteria grazing activity was 10.07 g. The approximated quantity of N released due to ciliates activities was 0.1 g per potato (Table 1). This quantity is about 30.30% of the amount of nitrogen that is artificially applied (0.33 g). Ciliates not only contain amounts of carbon and nitrogen, but also phosphorus and other essential elements [27]. They are abundant, cost-efficient, environmentally friendly, and sustainable [28]. These findings prove that ciliates are good candidates for green organic fertilizers. In practice, we need to consider ciliate diversity, special plant–soil conditions, and the interaction between microorganisms. An effective direct estimation method for determining the precise carbon and nitrogen contributions of ciliates still requires further elucidation.

### 3.2. Driving Factors of Potato Rhizosphere Ciliate Community

**Figure A1:**
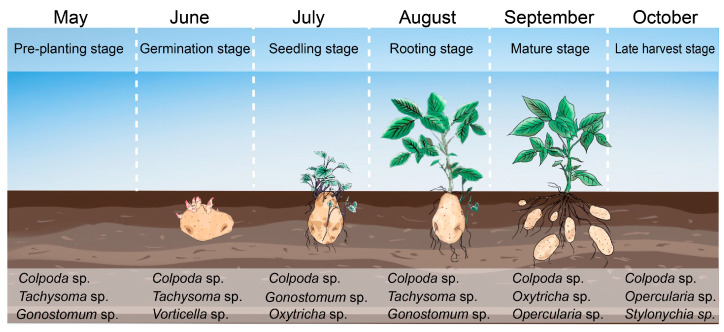
The dominant ciliate species at different stages.

The composition and structure of ciliate communities are crucial for their ecological functions [29], which are influenced by various factors, including plants [28], soil properties [30], climate factors [31], and microorganism interactions [32]. In this study, the dominant species of ciliate community showed a succession process driven by potato growth (Figure A1). This may be related to changes in the root exudate, which attract different beneficial ciliates to colonize the rhizosphere to enhance nutrient cycling and soil structure formation [32]. Among these dominant ciliates, *Colpoda* sp. remained continuously dominant at all growth stages. It suggests that *Colpoda* sp. might be a core ciliate for the potato rhizosphere microorganism community.

**Figure A2:**
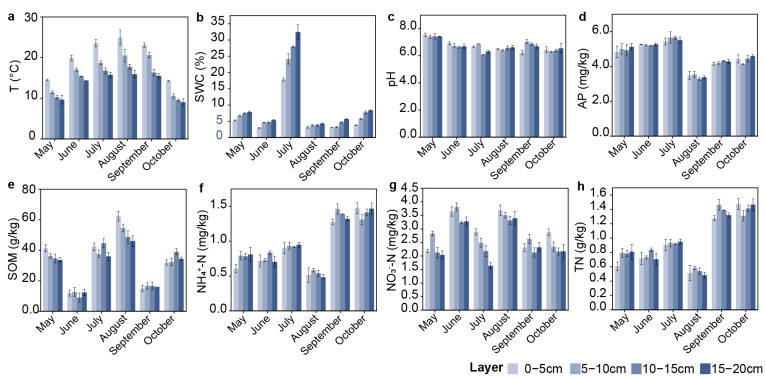
Physiochemical properties of soil from May to October: (**a**) temperature (T), (**b**) soil water content (SWC), (**c**) pH, (**d**) available phosphorus (AP), (**e**) soil organic matte (SOM), (**f**) ammonium nitrogen (NH_4_^+^-N), (**g**) nitrate nitrogen (NO_3_^−^-N), and (**h**) total nitrogen (TN).

**Figure A3:**
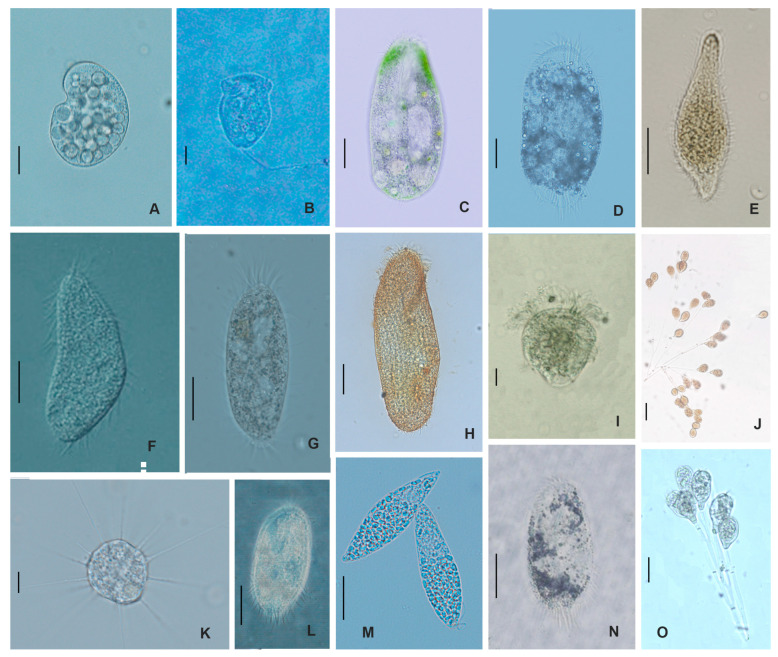
Photomicrographs of some rhizosphere protozoa. (**A**) *Colpoda* sp., (**B**) *Vorticella* sp., (**C**) *Pauroleptus* sp., (**D**) *Stylonychia* sp., (**E**) *Litonotus* sp., (**F**) *Gonostomum* sp., (**G**,**N**) *Oxytricha* sp., (**H**) *Parakahliella* sp., (**I**) *Strongylidium* sp., (**J**) *Zoothamnium* sp., (**K**) *Actinophrys* sp., (**L**) *Tachysoma* sp., (**M**) *Astasia* sp., and (**O**) *Opercularia* sp. Scale bars = 20 μm.

On the other hand, physicochemical factors clearly have an influence on the ciliate community. The authors of studies have found that ciliate communities are more sensitive to nitrogen than other microorganisms in diverse agricultural soils are [4,30]. Our results proved that *Colpoda* sp. and most ciliates are significantly and positively correlated with NH_4_^+^-N, SOM, and SWC. *Colpoda* sp. and *Oxytricha* sp. are negatively correlated with changes in NO_3_^−^-N. The species and number of ciliates were highest at the seedling stage (July), which is attributed to the highest SWC (Figure A2b). This suggested that SWC and nitrogen should be the environmental drivers of the ciliate community. They should be considered in farm management and potato cultivation.

Accurate identification and classification are pivotal to studying the diversity of ciliates [33]. Molecular techniques, such as PCR amplification and the sequencing of specific genetic markers, such as the 18S rRNA gene, have been employed to achieve the more precise and reliable identification of ciliate species. Based on high-throughput sequencing and the 18S rRNA gene, it has been revealed that the quantity and role of ciliates in promoting material circulation in soil were underestimated, and many more potential ciliate species remained uncultured or unidentified [1,34]. The more accurate and efficient identification of ciliate species would lead to a deeper and fuller understanding of the nutritional contributions of ciliates [35].

## 4. Materials and Methods

### 4.1. Sample Collection and Species Identification

All samples were obtained from a monoculture field of potatoes located in Heilongjiang Province (China N 45°59′38″, E 126°37′57″). The annual amount of precipitation in this area is 400–800 mm, and the annual average temperature is 0–10 °C. One month before sowing (May), the amount of fertilizer used was about 40–50 kg per acre. The ratio of diamine, potassium sulfate, and urea was 2:2:1, and the nitrogen contents of diamine and urea were 18% and 46%. 

Soil samples were collected based on the farming stages of the potatoes: the pre-planting stage (May), germination stage (June), seedling stage (July), rooting stage (August), mature stage (September), and late harvest stage (October) [36]. At each sampling stage, 2 kg of rhizosphere soil was sampled from area 3 cm away from the plants at one of the four depths from top (0–5 cm, 5–10 cm, 10–15 cm, and 15–20 cm) using the five-point sampling method [37].

The non-flooded Petri dish method [38] was performed in Petri dishes at room temperature. The suspension was observed under a microscope from the 1st to the 15th days of culturing, and the observed protozoa were identified via in vivo observation. The protargol method was used to identify the group of protozoa [39]. Silver carbonate [40,41] and Chatton–Lwoff silver nitrate stains [42] were also used to classify and identify certain kinds of protozoa, respectively.

### 4.2. Physicochemical Soil Analyses

Bulk soil was obtained at the same sampling location at four soil layers, which correspond to the protozoa samples. The physicochemical properties included the soil water content (SWC), concentrations of soil organic matters (SOM), total nitrogen (TN), available phosphorus (A-P), ammonium nitrogen (NH_4_^+^-N), nitrate nitrogen (NO_3_^−^-N), soil pH, and temperature (T). All these parameters were detected according to the Chinese National Standard (GB 15618-1995).

### 4.3. Estimation of Carbon and Nitrogen Contribution Rates

We used the following parameters for calculations: soil density—1.3 g/cm^3^; bacteria biovolume—0.65 μm^3^ [43]; each cm^3^ of bacterial biovolume had 0.22 g C [44]; bacterial grazing rate by ciliates was 523 ind./h respectively [45]; the rhizosphere for potatoes covered a 30 × 60 cm^2^ projection area. According to the abundance and cell size of ciliates, over six months (Table A1), the carbon and nitrogen flows per planted potato had a 30 × 60 × 15 cm^3^ projection area. We also used C/N ratios for typical bacteria and ciliate of 5.2:1 and 6.8:1, respectively [45], and assuming 100% carbon catabolism, the fixation of 1 g of C to protozoa cells released 0.045 g N to the soil. The weight increases between the germination stage, seedling stage, rooting stage, and mature stage were 40 g, 480 g, 550 g, and 200 g, respectively. The carbon contents used in the planted potatoes were 40% of the weight, and the nitrogen contents used were 10% of those of carbon contents. 

We calculated the carbon and nitrogen flows of ciliates according to Equation (1).

(1) $$Carbon\left(or\;Nitrogen\right)flow=Ingestion-Egestion-Respiration$$

where *ingestion* is the total amount of *carbon* (or *nitrogen*) that ciliates feed on bacteria from outside; *egestion* is the amount of unassimilated *carbon* (or *nitrogen*) by ciliates; *respiration* is the amount of carbon (or *nitrogen*) metabolized by ciliates during respiration. 

The calculated the ciliate biomass using Equation (2).

(2) $$Biomass=444.5pg\;C+(V{\mu m}^{3}\times 0.053pg\;C)$$

Ciliate body volume (*V*) was computed via measuring the linear dimensions of body parts under a microscope, followed by best geometric approximation [46]. These V data were converted into carbon (*C*) or nitrogen (*N*) using conversion factors: 1 μm^3^ *V* = 0.071 pg *C* = 0.0185 pg N for respiration data and 1 pg WM = 0.20 pg DM for egestion data [47].

The contribution rate was calculated using Equation (3).

(3) $$Contribution\;rate=\frac{F_{c}}{F_{p}}\times 100\%$$

where *F_c_* is the carbon (or nitrogen) flow of ciliates, and *F_p_* is the carbon (or nitrogen) flow of potatoes.

### 4.4. Statistics Analysis

In this study, excel was used to sort out and summarize the original data. The contributions of ciliates to three biodiversity indices and BIO-ENV analysis were calculated using PRIMER 7.0 multivariate analysis software. Origin 2021 was used to draw the variation pattern of environmental factors, the accumulation histogram of ciliate abundance, and the box plot of the ciliate diversity index. The community diversity indices included the Shannon–Wiener index (H′), Pielou evenness index (J), and Margalef species richness index (d) [48]. 

The correlation heat map between ciliate abundance and physicochemical properties was drawn using R language’s (version 3.3.1) built-in ‘pheatmap’ package. Pairwise comparisons of physicochemical properties are shown with a color gradient denoting Spearman’s correlation coefficients. Community diversity was related to each environmental factor identified using Mantel tests. Edge width corresponds to Mantel’s r statistic for the corresponding distance correlations, and edge color denotes the statistical significance based on 9999 permutations [43]. Mantel test, Principal Component Analysis (PCA), Non-metric Multidimensional Scaling (NMDS), and Redundancy analysis (RDA) were performed using Tutools platform (https://www.cloudtutu.com) (accessed on 3 March 2023).

## 5. Conclusions

The authors of the current study, for the first time, revealed the spatial–temporal distributions of ciliates in the potato rhizosphere. The diversity of ciliates was higher in the top soil and increased as the potatoes grew, but they were more abundant in the deep soil, and the number decreased as the potatoes grew. The highest species numbers and the most abundant rhizosphere ciliates appeared in July (seedling stage). According to the abundance during the six growth stages, five species were selected as core species, and *Colpoda* sp. was the dominant species at all growth stages. NH_4_^+^-N, NO_3_^—^N, and SWC greatly influenced the abundance of ciliates. This study established a method for estimating the contribution of carbon and nitrogen by ciliates to crops. The average contribution rates were 30.57% for carbon and 23.31% for nitrogen. The highest C/N contribution rates reached 94.36% and 72.29%, respectively, at the seedling stage. These results suggest that ciliates should be good candidates organisms for organic fertilizers. These new discoveries should be considered in farm management and potato cultivation.

## Figures and Tables

**Table 1:** Estimating contribution rates of rhizosphere soil ciliates on carbon and nitrogen flows for potatoes.

Nutrient Element	Parameters (g/Plant)	May	June	July	August	September	October	Avg.
Carbon	Ingestion	144.73	2.24	183.22	17.83	6.23	7.93	60.36
	Egestion	3.01	0.16	1.17	0.20	0.04	0.05	0.77
	Respiration	2.26	0.12	0.88	0.15	0.03	0.04	0.58
	Carbon flow	139.46	1.96	181.17	17.47	6.16	7.83	59.01
Carbon contribution rate (%)		-	12.26	94.36	7.94	7.71	-	30.57
Nitrogen	Ingestion	27.83	0.43	35.23	3.43	1.2	1.52	11.61
	Egestion	0.78	0.04	0.30	0.05	0.01	0.01	0.20
	Respiration	0.59	0.03	0.23	0.04	0.01	0.01	0.15
	Nitrogen flow	26.46	0.36	34.7	3.34	1.18	1.50	11.26
Nitrogen contribution rate (%)		-	8.96	72.29	6.07	5.90	-	23.31

## Data Availability

All data are presented in the article.

## Appendix A

**Table A1:** Ciliates abundance, biomass, and body size during six growth stages of potatoes.

Species	Ciliate Abundance (ind./g)						Body Length (μm)	Body Width (μm)	Volume (×10^4^ μm^3^)	Biomass (×10^−9^ g/ind.)
	May	June	July	August	September	October				
*Colpoda* sp.	66,000.00	891.00	90,500.00	5600.00	1925.00	2340.00	81.46	61.31	6.40	3.84
*Tachysoma* sp.	4200.00	160.00	1250.00	1760.00	127.00	880.00	120.00	48.46	8.92	5.17
*Oxytricha* sp.	2600.00	17.90	2860.00	150.00	800.00	120.00	89.08	38.85	5.14	3.17
*Gonostomum* sp.	4000.00	0.00	2940.00	1410.00	11.90	540.00	85.15	41.54	5.04	3.12
*Vorticella* sp.	422.60	131.00	272.00	127.00	18.00	50.00	72.46	26.00	3.05	2.06
*Spathidium* sp.	75.00	2.90	520.00	55.00	33.50	47.00	79.92	30.00	3.80	2.46
*Dileptus* sp.	194.00	5.90	11.90	180.00	0.00	9.20	82.15	32.15	4.10	2.62
*Stylonychia* sp.	47.00	0.00	281.00	216.90	8.30	24.00	112.77	51.15	8.44	4.92
*Pauroleptus* sp.	25.00	0.00	16.00	0.00	6.40	2.50	68.92	33.31	3.28	2.18
*Parakahliella* sp.	25.00	0.00	13.50	0.00	27.00	21.00	78.00	33.38	3.90	2.51
*Tetrahymena* sp.	370.00	0.00	242.00	98.00	224.00	64.00	46.00	17.62	1.27	1.12
*Strongylidium* sp.	157.00	0.00	32.50	20.00	18.00	4.90	142.85	59.77	12.90	7.28
*Opercularia* sp.	101.00	0.00	0.00	0.00	170.00	182.00	51.62	23.62	1.78	1.39
*Litonotus* sp.	0.00	0.00	53.50	18.00	0.00	0.00	80.00	33.38	4.04	2.59
*Zoothamnium* sp.	0.00	0.00	21.70	0.00	0.00	0.00	110.54	39.69	7.09	4.20
Total biomass (×10^−4^ g/plant)	9.07	0.47	3.53	0.61	0.12	0.16				

**Table A2:** Cumulative contribution of potato rhizosphere ciliates over six months.

Species	May			June			July			August			September			October		
	N	%	Rank	N	%	Rank	N	%	Rank	N	%	Rank	N	%	Rank	N	%	Rank
*Colpoda* sp.*	128.38	50.2	1	14.68	48.68	1	148.3	52.84	1	36.88	30.82	1	21.86	33.41	1	23.98	29.85	1
*Tachysoma* sp.*	32.38	12.69	2	6.26	21.69	2	17.61	6.69	4	20.92	18.85	2	5.59	8.37	6	14.76	18.93	6
*Gonostomum* sp.*	30.46	9.92	3	0	0	-	27.1	10.83	2	18.74	17.06	3	1.7	2.44	9	11.5	14.19	9
*Oxytricha* sp.*	25.24	9.06	4	2.02	6.26	4	26.7	10.45	3	6.09	5.33	6	14.1	21.66	2	5.26	5.68	8
*Tetrahymena* sp.	9.59	3.62	5	0	0	-	7.74	2.94	8	4.86	4	8	6.93	7.85	8	3.97	5.01	14
*Vorticella* sp.*	9.68	2.91	6	5.51	16.7	3	8.24	3.24	7	5.57	4.7	7	2.07	2.84	14	3.49	4.31	10
*Dileptus* sp.	6.95	2.65	7	1.21	4.44	5	1.65	0.53	14	6.68	6.01	5	0	0	-	1.51	1.98	4
*Strongylidium* sp.	6.24	2.32	8	0	0	--	2.81	1	10	2.22	1.94	10	2.08	2.86	10	1.09	1.33	12
*Opercularia* sp.*	5.02	1.95	9	0	0	-	0	0	-	0	0	-	6.32	8.43	3	6.6	7.79	2
*Spathidium* sp.	4.31	1.61	10	0.8	2.24	6	11.37	4.37	5	3.69	3.21	9	2.89	4.61	7	3.43	4.69	13
*Stylonychia* sp.*	3.42	1.31	11	0	0	-	8.38	3.35	6	7.32	6.42	4	1.43	2.11	4	2.39	2.78	3
*Pauroleptus* sp.	2.48	0.91	12	0	0	-	1.99	0.76	12	0	0	-	1.26	1.89	12	0.78	0.95	5
*Parakahliella* sp.	2.46	0.85	13	0	0	-	1.83	0.69	13	0	0	-	2.54	3.51	5	2.22	2.51	7
*Litonotus* sp.	0	0	-	0	0	-	3.66	1.45	9	2.07	1.65	11	0	0	-	0	0	-
*Zoothamnium* sp.	0	0	-	0	0	-	2.31	0.86	11	0	0	-	0	0	-	0	0	-

Note: N indicates average abundance; % indicates cumulative contribution rate. * Top three important contributors at each stage.

**Table A3:** Ciliate diversity indexes in four layers of soil during six growth stages of potatoes.

	H’	J’	D
May 1	1.78	0.70	2.14
May 2	1.76	0.69	2.16
May 3	1.77	0.69	2.15
May 4	1.79	0.70	2.15
June 1	1.45	0.81	1.44
June 2	1.49	0.83	1.57
June 3	1.31	0.73	1.43
June 4	1.28	0.71	1.43
July 1	1.79	0.68	2.38
July 2	1.76	0.67	2.35
July 3	1.57	0.59	2.28
July 4	1.56	0.59	2.29
August 1	2.09	0.87	2.13
August 2	2.01	0.84	2.12
August 3	1.95	0.81	2.10
August 4	1.96	0.82	2.08
September 1	2.08	0.84	2.64
September 2	2.03	0.82	2.56
September 3	2.06	0.83	2.59
September 4	1.99	0.80	2.62
October 1	2.15	0.84	2.76
October 2	2.17	0.85	2.81
October 3	2.12	0.83	2.71
October 4	2.05	0.80	2.66

Note: The Shannon-Wiener index (H’), Pielou evenness index (J’), and Margalef species richness index (D). The numbers in the figure represent different layers of soil 1: 0–5 cm; 2: 5–10 cm; 3: 10–15 cm; 4: 15–20 cm.

**Table A4:** Biota–environment (BIOENV) analysis of soil physicochemical properties.

Rank	*R*	Environmental Variables	*p*
1	0.677	NH_4_^+^-N, SOM, TN, pH	0.01
2	0.677	NH_4_^+^-N, SOM, TN	0.01
3	0.673	NH_4_^+^-N, AP, SOM, pH	0.01
4	0.672	NO_3_^−^-N, NH_4_^+^-N, SOM, TN	0.01
5	0.672	NO_3_^−^, NH_4_^+^-N, SOM, TN, pH	0.01
6	0.671	NH_4_^+^-N, AP, SOM, TN, pH	0.01
7	0.671	NO_3_^−^-N, NH_4_^+^-N, AP, SOM, TN, pH	0.01
8	0.670	NH_4_^+^-N, AP, SOM, TN	0.01
9	0.669	NO_3_^−^-N, NH_4_^+^-N, AP, SOM, TN	0.01
10	0.668	NH_4_^+^-N, AP, pH	0.01

*R* indicates spearman correlation coefficient.
